# Association between the Obstructive Sleep Apnea and Cephalometric Parameters in Teenagers

**DOI:** 10.3390/jcm12216851

**Published:** 2023-10-30

**Authors:** Gayane E. Manrikyan, Izabella F. Vardanyan, Marina M. Markaryan, Mikayel E. Manrikyan, Elen H. Badeyan, Anna H. Manukyan, Mariana A. Gevorgyan, Samson G. Khachatryan

**Affiliations:** 1 Department of Therapeutic Dentistry, Yerevan State Medical University (YSMU), Koryun Str. 2, Yerevan 0002, Armenia; marmiga@mail.ru (M.M.M.);; 2 Department of Pediatric Dentistry and Orthodontics, Yerevan State Medical University (YSMU), Koryun Str. 2, Yerevan 0002, Armenia; iza.vard67@gmail.com (I.F.V.); dr.manrikyan@mail.ru (M.E.M.);; 3 “Somnus” Neurological Clinic, Department of Neurology and Neurosurgery, National Institute of Health, Yerevan 0051, Armenia; drsamkhach@gmail.com

**Keywords:** orthodontic(s), polysomnography, occlusion, cephalometry, sleep apnea, sleep dentistry

## Abstract

Background: OSA is a common problem in children and adolescents. Angle class II malocclusion, a tendency toward the vertical type of growth, causes a decrease in the volume of the oral air space, increasing the risk of OSAS. The aim of this study was to evaluate the relationship between cephalometric and OSA parameters, to develop collaborative approaches between orthodontists and somnologists in the treatment of adolescents with OSA. Methods: We analyzed data from 41 adolescents with OSA. Their mean age was 15.8 ± 1.08 years. Orthodontic and polysomnographic examinations of patients were conducted. Statistical analysis was performed in SPSS 19.0.0. Results: Most often in patients with distal occlusion, a violation of the harmony in the development of the dental system was observed. The sagittal incisive fissure, characteristic of a distal occlusion, was absent due to the palatal inclination of the upper incisors in 25 (60.98%) patients. The SNB was 79.4 ± 3.1°, indicating a distal position of the mandible relative to the anterior cranial base. The SNA exceeded the normal value, which is one of the prerequisites for mandibular retrognathia. The ANB angle was 4.3 ± 1.9°. Tonsillar hypertrophy affected 6 patients, 21 had adenoid hypertrophy, and 3 had both of them. Movements of the masticatory muscles during sleep were recorded in 22.0% of patients. Conclusion: To improve the quality of diagnosis and treatment of OSA, a multidisciplinary approach is needed that will correct the processes of child growth and development.

## 1. Introduction

Dental sleep medicine is the field of dental practice that deals with the management of sleep-related breathing disorders, which include obstructive sleep apnea (OSA) in adults and children. Obstructive sleep apnea (OSA) is a sleep disorder in which breathing is repeatedly interrupted during sleep [1,2]. Often its duration is 20–30 s; in severe cases, it can reach 2–3 min and occupy up to 60% of the total duration of the nocturnal sleep, with daytime sleepiness and disruption of sleep structure, deterioration of memory and intelligence, complaints of decreased performance, and constant fatigue leading to OSA syndrome (OSAS). It is a common problem in children and adolescents, affecting up to 47% of overweight adolescents [3].

Severe disruption of sleep structure and decreased blood oxygenation influence the emergence of neurocognitive development problems in adolescents with OSA, including decreased attention, memory, hyperactivity, and increased aggression [1,2]. 

It has been proven that adolescents with sleep disorders demonstrate a lower level of executive functions, which correlates with the predominance of shallow stages of sleep over deep stages and more frequent awakenings reducing the quality of sleep [4]. 

Upper airway (UA) narrowing and its subsequent collapse are the leading mechanism for the occurrence of OSAS episodes [5]. Relaxation of pharyngeal muscles during sleep leads to increased mobility of the airways’ walls, and further reduction in muscle tone leads to complete or partial collapse of the airways [6,7,8,9]. It has been recorded that upon entering the slow-wave sleep phase, the volume of the UA can be reduced by up to 40%. Factors that may play a role in the pathogenesis of OSAS are changes in oropharyngeal muscle activity that occur during sleep, poor responsiveness of the genioglossus muscles to negative pressure in the pharynx, a low respiratory arousal threshold, and a hypersensitive ventilatory control system [10]. Moreover, genioglossus muscle activity is significantly reduced during rapid eye movement (REM) sleep, especially during REM sleep coinciding with the onset of REM hypopnea/obstructive apnea [11]. The resulting hypoxemia causes activation of the nervous system and brief awakening. Mouth breathing during sleep lengthens and narrows the UA, which, in turn, can exacerbate the severity of OSA [12,13]. Proliferation of lymphoid tissue impedes the passage of airflow through the respiratory tract, narrowing the lumen of the pharyngeal space [14].

Angle class II malocclusion, a tendency toward the vertical type of growth, and a posterolateral crossbite directly cause a decrease in the volume of the oral air space, increasing the risk of OSAS [15,16,17]. A timely detected tendency to develop dentoalveolar deformities can be resolved with properly selected orthodontic treatment. According to the literature analysis, both upward and anterior displacement of the hyoid bone appears as an adaptive factor of the airways against obstruction [17,18]. The area in which obstruction of the UA occurs during sleep is most often located at the level of the soft palate and the root of the tongue or epiglottis, i.e., in the lower part of the nasopharynx and oropharynx, due to narrowing of the oropharynx with distalization of the mandible and micrognathia, especially in the setting of obesity [19,20].

OSA can have a number of negative consequences for teenagers, including sleepiness, fatigue, difficulty concentrating, and impaired cognitive function. It can also increase the risk of developing other health problems, such as high blood pressure, heart disease, and stroke. These disorders are reversible with timely treatment, which makes early diagnosis and treatment of OSAS especially important. The main clinical manifestations of mouth breathing occur in the craniofacial structures. Those who breathe through their mouth often suffer from malocclusions and craniofacial bone abnormalities. Chronic muscle tension around the mouth can lead to a widening of the craniovertebral angle, a posterior positioning of the mandible, and a narrowing of the maxillary arch. In addition to malocclusion, mouth breathers often experience chronic gingivitis, bruxism, periodontitis, candida infections, and halitosis [21,22,23].

The resolution of these anomalies in cases that do not require surgical intervention is carried out by an orthodontist. Dental treatment options may vary depending on the patient’s age and the cause of apnea. For pediatric patients, treatment may include non-surgical maxillary expansion and orthodontic functional appliances [24,25]. Many physicians and dentists are unaware of the role dentistry, particularly orthodontics, may play in the interdisciplinary management of these disorders.

The relationship between bad habits, mouth breathing, and malocclusion is an important issue in terms of prevention and early treatment of craniofacial growth disorders. While bad habits may interfere with tooth positioning and normal skeletal growth patterns, on the other hand, UA obstruction resulting in mouth breathing alters the craniofacial growth pattern causing malocclusion [26,27]. Therefore, it is necessary to promptly address these etiological factors of malocclusion in order to prevent its development or worsening, and, if it has already developed, to correct it through early orthodontic treatment to promote normal skeletal growth [28]. 

Despite the high prevalence of OSA in teenagers and its negative consequences, there is a lack of research on the effectiveness of collaborative approaches between orthodontists and sleep physicians in the treatment of OSA in this population, aimed at maximizing the benefits and minimizing the side effects in the treatment of teenagers with OSA.

The aim of the study was to evaluate the relationship between cephalometric and OSA parameters, to develop collaborative approaches between orthodontists and somnologists in the treatment of adolescents with OSA.

## 2. Materials and Methods

We analyzed data from 41 adolescents with OSA examined at Dental Clinic No. 1 of Yerevan State Medical University and the “Somnus” Sleep Laboratory, Yerevan, Armenia. Patients consulted an orthodontist regarding orthodontic problems. When collecting an anamnesis, complaints about snoring were revealed, and in the life history, there was a violation of the nasal flow and hypertrophy of the tonsils. Therefore, patients were referred to a sleep center for polysomnographic studies.

Their mean age was 15.8 ± 1.08 years, and 35 (85.4%) were males. All participants belonged to the Indo-European race and to the ethnic group of Armenians. The socioeconomic status of the participants did not differ significantly; middle and high school children took part in this study. All participants and their parents provided written informed consent before being included in this study. This study was conducted in accordance with the Declaration of Helsinki and received approval from the Ethics Committee of Yerevan State Medical University N. 10-15 (19 June 2014). 

The examination included a survey, clarification of complaints, history of the development of underlying disease, and a clinical examination. The latter played a crucial role in the screening process. The examination was carried out in a dental chair under artificial light. 

The following eligibility criteria were applied:

Inclusion criteria:

○ Nasal breathing disorder;
○ Dentofacial anomalies.

*Exclusion criteria:*

○ Bad habits;
○ Refusal of the examination (conducting a cephalometric or polysomnographic study);
○ Ongoing orthodontic treatment;
○ Taking medications that affect sleep;
○ Renal failure;
○ Neuromuscular diseases;
○ Neurological disorders;
○ Uncontrolled diabetes;
○ Heart failure;
○ Thyroid disease;
○ Any other uncontrolled disease.

The orthodontic examination consisted of an initial assessment of maxillofacial features on site and referral for a cephalometric X-ray examination using a Planmeca ProMax Type 3D+ (Planmeca) set at 12 mA, 90 kV, and an exposure time of 0.30 min. The results were analyzed using Romexis Viewer 5.4.1 (Planmeca). The position of many cephalometric landmarks during the study directly depends on the orientation of the head in space. Natural head position (NHP) is the most correct physiological and anatomical orientation for assessing the harmony of the face and the position of the jaws and teeth. To obtain reliable results, in this study, all images were recorded with the patient’s head fixed in the cephalostat.

SNA (angle between sella, nasion, and subspinale point A; degrees), SNB (angle between sella, nasion, and supramentale point B; degrees), and ANB (angle between the maxilla and the mandible; degrees) were used to determine the position of the maxilla and mandible with respect to the anterior cranial base (sella–nasion, SN line) and the respective skeletal class. 

Cranial base angle (BaSN; degrees) and angle formed between GoAr and GoGn lines (ArGoGn; degrees) revealed the growth direction.

Maxillomandibular plane angle (MxPl/MnPl; degrees) represented the relationship of the upper and lower jaws. 

The key linear cephalometric parameters are:

AH-C3H (anterior–superior point of the hyoid bone to third cervical vertebra horizontal distance, mm);

MPH (vertical distance from mandibular plane to hyoid bone, mm);

EbTt (vertical position of the tongue, mm);

U1NA (upper incisor inclination, degree);

L1NB (lower incisor inclination, degree);

Overjet (sagittal gap—distance from the vestibular surface of the lower jaw incisors to the palatal surface of the upper jaw incisors, mm);

Overbite (vertical gap—distance from the cutting edge of the central incisor of the upper jaw to the cutting edge of the central incisor of the lower jaw, mm);

PAS1 (upper pharyngeal airway space; between the PPW and the posterior line of the soft palate, at the level of the palatal plane, mm);

PAS2 (middle pharyngeal airway space; between the PPW and the lower edge of the uvula, mm);

PAS3 (lower pharyngeal airway space; between the PPW and the base of the tongue, at the level of the mandibular line, mm);

PASmin (minimal pharyngeal airway space at the epiglottis, mm) [29]. 

Cephalometric analysis was performed manually twice by different individuals to exclude the factors of human mistakes, and we included linear and angular parameters.

Patients were referred to the sleep disorder center for diagnostic testing utilizing a portable respiratory polygraph (Embletta X10, Embla) or nocturnal polysomnography (Embla N7000, Embla). Polysomnography parameters were calculated using SomnoLogica software (v. 5.1.1, Embla). 

Polysomnography is based on recording vital signs during sleep: bioelectrical activity of the brain (electroencephalography, EEG), eye movements (electrooculogram, EOG), activity of the chin muscles (electromyography, EMG), electrocardiogram (ECG), and limb movements. It is mandatory to record airflow and respiratory effort, blood oxygen saturation, sound phenomena (snoring), and body position in bed.

The severity of OSA for patients in a given age group was classified based on the Apnea–Hypopnea Index (AHI = number of apneas + hypopneas/total sleep time), categorized as follows [30,31]:

Mild OSA: AHI ≥ 5 to < 15 events per hour;
Moderate OSA: AHI ≥ 15 to < 30 events per hour;
Severe OSA: AHI ≥ 30 events per hour.

Minimum oxygen saturation should also be considered in the clinical assessment of the severity of OSA, although generally accepted classifications for the severity of oxygen desaturation are lacking.

Statistical analyses were performed using SPSS software (v. 19.0.0.0, Statistical Package of Social Sciences; IBM Corp). Descriptive statistics were performed expressing continuous data as means with standard deviations (SDs), whereas categorical data were expressed as frequencies and percentages. Comparisons were evaluated using Student’s *t*-test. Correlations between cephalometric and polysomnographic parameters were calculated using Pearson’s correlation test. A *p*-value less than 0.05 was considered statistically significant.

## 3. Results

Dentoalveolar and skeletal changes observed against the background of oral breathing affect the psychological state of children. The presence of a sagittal gap, significant protrusion of the incisors, and expressed narrowing of the dentition of the upper and lower jaw are accompanied by the formation of increased anxiety in growing patients, which is reflected in the levels of social and psychological adaptation of children in society.

**Figure 1:**
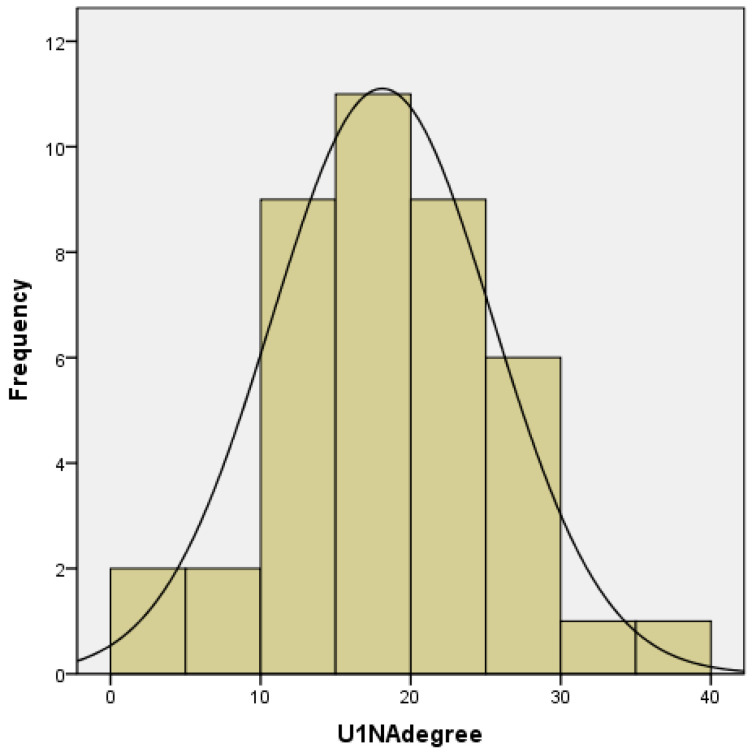
Inclination range of the upper incisors.

**Figure 2:**
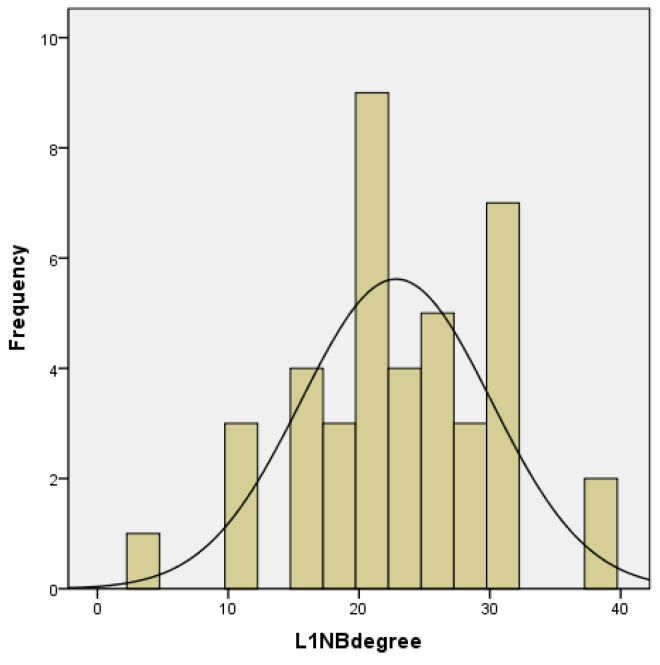
Inclination range of the lower incisors.

The morphological characteristics of this anomaly in patients of this group had significant differences. The sagittal incisive fissure, characteristic of a distal occlusion, was absent due to the palatal inclination of the upper incisors in 25 (60.98%) patients. Cephalometric assessment of upper incisor inclination showed a range of 36° for U1NA/deg (mean 18.11°, SD 7.365°). The lower incisor inclination ranged within 38° (mean 22.8°, SD 7.28°). In 14 (34.15%) cases, protrusion of the upper incisors was observed (Figure 1 and Figure 2). 

Thus, most often in patients with distal occlusion and retrusion of the upper incisors, a violation of the harmony in the development of the dental system was observed. The SNB angle was 79.4 ± 3.1° (*p* < 0.001), indicating a distal position of the mandible relative to the anterior cranial base. 

An increase in the average sella–nasion to palatal plane (SNPP) angle to 8.38 ± 3.39° indicated clockwise rotation of the maxilla. The SNA angle exceeded the normal value (83.5 ± 3.03°), which is one of the prerequisites for mandibular retrognathia. 

The ANB angle was 4.3 ± 1.9° (*p* < 0.001) (relationship of the upper and lower jaws), ArGoGn was 130.47 ± 8.99° (*p* < 0.001) (growth predictor), and AH-C3H was 32.69 ± 5.8° (*p* < 0.001). 

Overjet in patients was 4.01 ± 2.06, and overbite was 3.2 ± 2.1 (*p* < 0.001). Both parameters exceeded the norm.

Cephalometric parameters were not associated with age and were almost identical in males and females (Table 1). 

Considering that obesity (body mass index [BMI] ≥30 kg/m^2^) is one of the risk factors for the development of OSA in adolescents, we calculated the BMI for the subjects in this group. The average value was 22.49 ± 4.25 kg/m^2^ (*p* < 0.001). Five adolescents (12.2%) were found to have a BMI exceeding 30 kg/m^2^. 

Hypertrophy of the palatine tonsils, along with adenoids and deviation of the nasal septum, leads to obstruction of the upper respiratory tract with the development of skeletal and dentoalveolar changes at the level of the dentofacial apparatus. In total, 6 patients (14.6%) had tonsillar hypertrophy (TH), 21 (51.2%) had adenoid hypertrophy (AH), and 3 (7.3%) had both TH and AH. 

Movements of the masticatory muscles (bruxism) during sleep were recorded in 9 patients (22.0%). Oral breathing was detected in 14 patients (34.1%). The remaining 27 patients (65.9%) had a mixed type of breathing. 

## 4. Discussion

The craniofacial characteristics of children with habitual snoring have been studied extensively over the past decades and remain a subject of ongoing debate. Although it is not possible to identify a direct relationship between the cause of UA obstruction and its impact on craniofacial growth, mouth breathing may be associated with changes in the position of the orofacial muscles and the mandible, affecting functions such as chewing, swallowing, and phonation, consequently leading to occlusal and skeletal changes [13]. Many authors have reported that mouth breather children have increased total and lower anterior facial height, as well as a dolichofacial pattern rather than a mesofacial pattern. From this perspective, a long face and vertical facial growth appear to be closely related to mandibular rotation in these patients [18,28]. 

Although there is still a debate about whether mouth breathing is a cause or consequence of craniofacial changes and whether facial skeletal structure directly influences the development of adenoid size, our goal was to assess the direct correlation between airway patency and craniofacial characteristics, to better understand the relationship between snoring and cephalometric patterns. According to the obtained mean values, the length of the mandibular body was increased in size in 36.6% of patients, corresponded to the average normal statistical data (66.3 ± 1.2 mm) in 4.9%, and was reduced in 58.5%. Compared with the average normal statistical data, 9.8% of patients had a normal-sized maxilla, 80.4% had an enlarged one, and 9.8% a reduced upper jaw. 

When diagnosing and planning orthodontic treatment, it is essential to consider the type of growth of the jaw bones [15]. In our cohort of patients, 39.0% (16 adolescents) had a horizontal type of jaw growth, 9.8% (4 adolescents) had a neutral type, and 51.2% (21 adolescents) had a vertical type of growth. The data obtained confirm research from various authors [15]. The results in Table 2 demonstrate that the correlation of U1NA/deg to the sagittal position of the mandible (SNB) was significantly stronger than to the sagittal position of the maxilla (SNA). Similarly, lower incisor inclination (L1NB; mm) to SNB was significantly greater than to SNA. Thus, our results did not at all coincide with those of Knösel et al. (2008) [32], suggesting axial incisor inclination depends largely on the sagittal position of the corresponding jaw and to a lesser extent on the antagonistic jaw. 

The main parameter of the pharynx is the posterior pharyngeal airway space (PAS), i.e., the distance between the posterior pharyngeal wall (PPW) and the base of the tongue or soft palate. This area can be divided into three different levels: upper PAS (PAS1), middle PAS (PAS2), and lower PAS (PAS3). 

A moderate positive correlation (statistically significant) was revealed between the indicators of the lower PAS in the epiglottis area (PASmin) and the upper incisor inclination in mm (r = 0.34, *p* = 0.03), the vertical position of the tongue (EbTt: r = 0.361, *p* = 0.02), the lower facial height (ANS-Me: r = 0.331, *p* = 0.035), the maxillomandibular difference (MxMn-DF: r = 0.338; *p* = 0.03), and the C2SPC4SP index (r = 0.337, *p* = 0.031) (Table 3). 

The PAS1 parameter (in mm, characterizing the posterior upper airway space) demonstrated a moderate correlation with the mandibular plane (MP) parameter (r = 0.477; *p* = 0.002), whereas the PAS3 (lower airway space) parameter moderately correlated with the lower facial height (ANS-Me: r = 0.322, *p* = 0.04) and PNSEb parameter (r = 0.316; *p* = 0.044) (Table 3).

The patients with OSAS had a decrease in airway space, especially at the MP level (PAS3, Table 1). The hyoid bone position was also of great importance. In healthy people, the hyoid bone is located at the level of the C3-C4 cervical vertebrae, while in patients with OSA, it is usually located lower at the level of C4-C6 [17]. The vertical distance between the hyoid bone and the MP (MPH) in OSA patients was smaller (12.3 ± 4.3 mm) than normal. Through cephalometric analysis, an increase in the sagittal gap was revealed in the patients (4.01 mm), which was associated with a more posterior position of the lower jaw (SNB parameter was 79.4°) and shortening of the effective length of the lower jaw (Co-Gn parameter was 102.9 mm). About 59% (24 out of 41) of our patients had mandibular micrognathism and retrognathism in relation to the maxilla, which was consistent with certain findings from other authors [33,34,35]. Normally, inspiration induces a flow characterized by increased muscle activity of the pharyngeal dilators (more than 20 muscles) to minimize the constriction caused by negative intraluminal pressure. Among these muscles, the genioglossus muscle of the tongue is the most studied structure, given its pivotal role in expanding the pharynx, base of the tongue, and the PPW, resulting in a decreased size of the oropharynx. 

The obtained results may be used for decision making in the diagnosis and treatment of dentofacial anomalies and OSA. Parameters that have shown a correlation are recommended to be included in the daily diagnosis (i.e., in the cephalometric analysis) of orthodontic patients. This will enable the orthodontist to understand the need for further polysomnographic testing. An orthodontist should also be involved in the process of sleep examination in sleep centers. 

This study has potential limitations, and the main limitation is the small number of participants. By increasing the number of observations, it will be possible to obtain more representative data.

## 5. Conclusions

Diagnosing OSA in adolescents is often hindered by a lack of awareness among practicing physicians regarding sleep disorders. Changes in a child’s behavior, daytime hyperactivity, and poor academic performance are sometimes erroneously considered a normal part of adolescence and not recognized as potential symptoms of an underlying medical condition. The main complaint that should alert the physician is snoring. The orthodontist should meticulously collect the medical history, using special questionnaires and identifying symptoms of impaired sleep quality and changes in behavioral reactions. While carrying out the diagnostic protocol, it is essential to pay attention to the deterioration of the airway patency at the level of the nasopharynx and oropharynx. During orthodontic treatment of OSA, it is necessary to create an adequate volume of oral air space, as well as maintain the proper functioning of the myodynamic forces of the dentofacial system. A multidisciplinary integrated approach to recognizing symptoms and diagnosing OSA will allow ensuring timely prevention and enhancing the quality of treatment, as well as correcting the processes of growth and development of the child’s body.

All orthodontists should consider incorporating OSA screening as part of the routine patient history collection and physical examination. When an orthodontist has a clinical suspicion that a patient may have OSA, referral is warranted and a sleep medicine specialist is preferable. The final diagnosis of OSA must be made by a physician. Early diagnosis and treatment of OSA in teenagers are essential for their overall health and well-being, and orthodontists can play a vital role in this process. 

## Figures and Tables

**Table 1:** Distribution of the mean and SD values of all parameters under study (*n* = 41).

Parameters	Mean	Standard Deviation
SNA *	83.5	3.03
SNB *	79.4	3.13
ANB *	4.3	1.91
ArGoMe *	130.47	8.99
BaSN *	124.85	6.2
EbTt *	61.36	6.9
MPH *	12.3	4.3
NSH *	88.2	4.6
MnPl *	64.29	5.2
MxPL *	52.5	4.6
PAS1 *	17.2	3.2
PAS2 *	11.6	3.67
PAS3 *	4.8	2.68
PAS min *	3.7	1.37
AHC3H *	32.69	5.8

* *p* < 0.001.

**Table 2:** Pearson’s correlation coefficients (R) between upper and lower incisor inclination vs. sagittal–skeletal dimension and linear measurement (*n* = 41).

Parameters	SNA		SNB		ANB		MnPL	
	r	*p*	r	*p*	r	*p*	r	*p*
U1NA, mm	0.31	0.8	0.340	0.03 *	−0.271	0.09	0.378	0.015 *
U1NA, degree	−0.33	0.8	0.369	0.017 *	−0.267	0.07	0.441	0.004 *
L1NB, mm	−0.177	0.27	−0.325	0.038 *	0.357	0.022 *	0.272	0.086
L1NB, degree	0.269	0.08	0.056	0.3	0.286	0.07	0.079	0.6

* Correlation indicators are statistically significant.

**Table 3:** Coefficients of Pearson’s correlation of cephalometric and polysomnographic parameters with the apnea–hypopnea index.

Parameters	R	*p* *
MnPL	0.331	0.035
SGoNMe	−0.367	0.018
NSH	0.326	0.038
OSI	0.615	0.001
ODI	0.796	<0.001
SI	−0.482	0.001
AHI spine	0.885	<0.001
AHI nonspine	0.506	0.001
pulse	0.329	0.036

* Correlation indicators are statistically significant.

## Data Availability

The datasets used and analyzed during the current study are available from the corresponding author on reasonable request.
